# Treatment decision in a 4-year-old-boy with left ventricular outpouching after advanced hemodynamical flow evaluation with 4Dflow CMR: A case report

**DOI:** 10.3389/fped.2022.953770

**Published:** 2022-11-17

**Authors:** Kerstin Lagerstrand, Par-Arne Svensson, Linnea Andersson, Mats Synnergren, Annika Öhman, Magnus Petersson, Jan Sunnegardh, Frida Dangardt

**Affiliations:** ^1^ Department of Medical Physics and Biomedical Engineering, Sahlgrenska University Hospital, Gothenburg, Sweden; ^2^ Institute of Clinical Sciences, Sahlgrenska Academy, University of Gothenburg, Gothenburg, Sweden; ^3^ Department of Pediatric Radiology and Physiology, The Queen Silvia Children’s Hospital, Sahlgrenska University Hospital, Gothenburg, Sweden; ^4^ Department of Pediatric Cardiology, The Queen Silvia Children’s Hospital, Sahlgrenska University Hospital, Gothenburg, Sweden; ^5^ Institute of Medicine, Department of Molecular and Clinical Medicine, Sahlgrenska Academy, University of Gothenburg, Gothenburg, Sweden

**Keywords:** 4Dflow, 4D flow CMR, congenital heart disease, left ventricular outpouching, case report, phase contrast

## Abstract

**Background:**

The present study presents a diagnostic course for the characterization of a congenital left ventricular outpouching (LVO) in a 4-year-old boy with severe neonatal heart failure, evaluating the added value of cardiac magnetic resonance (CMR) 4Dflow.

**Case presentation:**

A boy, born at full term, presented with heart failure immediately after birth. Echocardiography showed dilated left ventricle with poor function and LVO was initially interpreted as an aneurysm. No infection, inflammation, or other cause for heart failure was found. With intensive medical treatment, the heart function returned to normal, and eventually, all medication was terminated. At follow-up, surgical treatment of the LVO was discussed but after CMR 4Dflow, a thorough evaluation of the function of the left ventricle as well as the LVO was possible and the LVO was determined a double-chambered left ventricle with a good prognosis.

**Conclusions:**

The present case demonstrates the clinical usability of CMR 4Dflow for improved decision-making and risk assessment, revealing advanced hemodynamic flow patterns with no need for operation.

## Introduction

Cardiac magnetic resonance (CMR) is used to map altered anatomy and its physiological effects prior to treatment decisions in children with congenital heart defects (CHDs). Recently, CMR 4D flow has been implemented in the clinic to allow detailed analysis of the blood hemodynamics (1), which may improve the diagnostic evaluation of patients with CHD.

**Figure 1:**
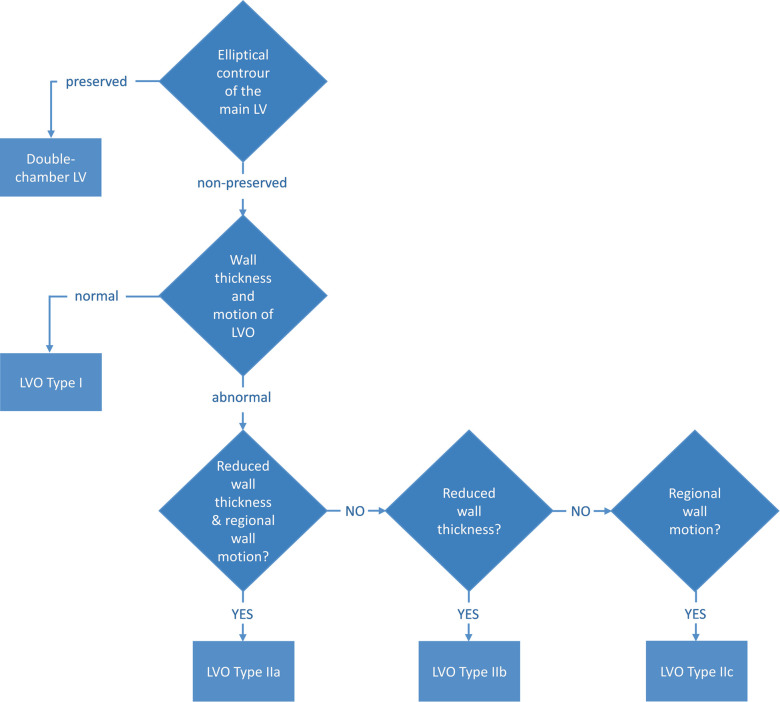
Classification of congenital LVO by Malakan Rad et al. (5). LVO, left ventricular outpouchings.

Congenital ventricular outpouchings are rare with an incidence of 1 per 200,000 live births (2–4). The anomaly is characterized by localized protrusions of the ventricular wall and presents a dynamic form where the outpouchings are most variable in the prenatal period and the first year after birth (3). As such, serial evaluations are recommended to evaluate the change in the characteristics of the outpouching (3). Such evaluation should include image-based methods, which can display relevant characteristics for classification of the outcome, i.e., geometry of the main ventricle, wall thickness, and regional wall motion (5) (Figure 1), as well as emptying of outpouching, size of connecting neck, and thrombus formation. Both echocardiography and CMR have been shown to contribute to the evaluation, but the novel 4Dflow method may add important functional information to the evaluation, which may improve the decision-making and risk assessment of patients with congenital ventricular outpouchings.

The present study presents a diagnostic course for the characterization of a congenital left ventricular outpouching (LVO), evaluating the added value of CMR 4Dflow.

## Case presentation

**Figure 2:**
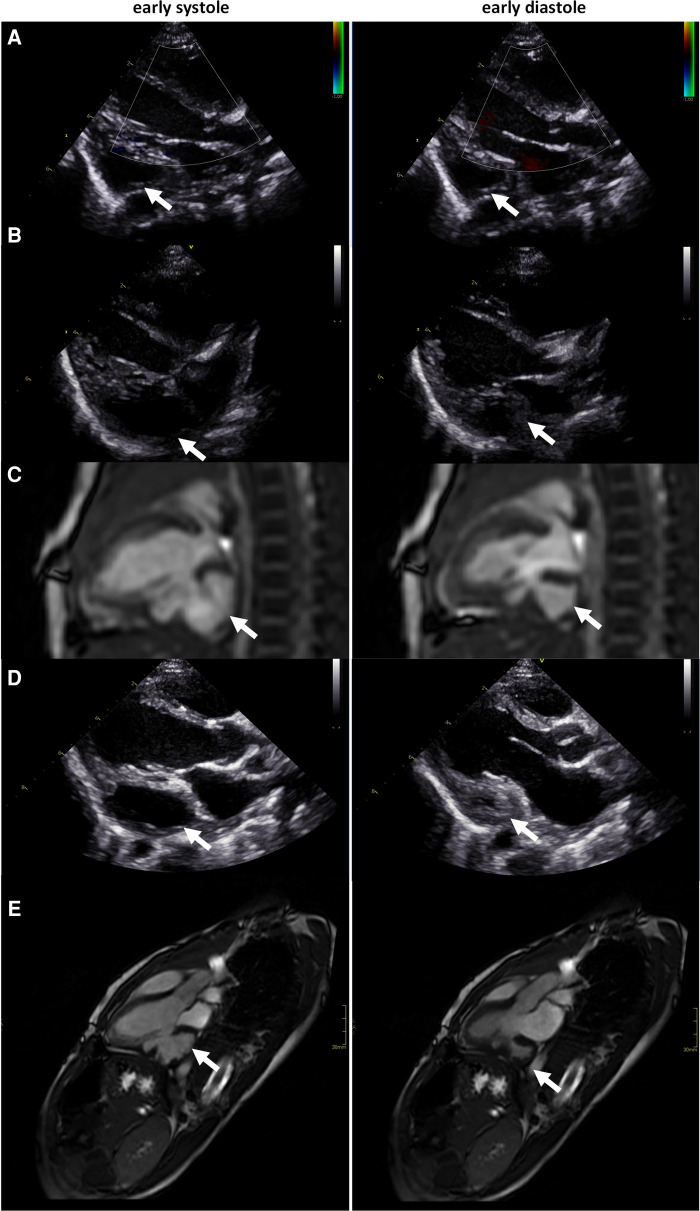
(**A**) Echocardiography at enrollment: (LVIDd/LVIDs: 2.1 cm/1.9 cm, FS 10%, LVEF 22%), (**B**) echocardiography at 14-day follow-up (LVIDd/LVIDs: 2.0 cm/1.5 cm, FS 27%, LVEF 55%), (**C**) CMR at 14-day follow-up (LVEDV: 10 ml, LVESV: 5 ml, LVEF 51%), (**D**) echocardiography at 4-year follow-up (LVIDd/LVIDs: 3.4 cm/2.0 cm, FS 40%, LVEF 73%), and (**E**) CMR at 4-year follow-up, displaying an ALS inferior of the left ventricle, determined to be an aneurysm (LVEDV: 40 ml, LVESV: 10 ml, LVEF 75%). CMR, cardiac magnetic resonance; ALS, aneurysm-like structure. LVIDd and LVIDs indicate left ventricular internal diameter end diastole and end systole, FS: fractional shortening, LVEF: left ventricular ejection fraction, LVEDV: left ventricular end diastolic volume, LVESV: left ventricular end systolic volume.

The patient is a boy, later diagnosed with autism, who was born at full term through a cesarean section due to slow progress. The birthweight was 4,105 g, length 52 cm, and Apgar score of 9 at 1 and 5 min. Immediately after birth, a fast deterioration with increased breathing rate and an Apgar score of 6 at 6 min due to severe heart failure followed. A loud systolic murmur was noted, and echocardiography revealed a dilated left ventricle with poor systolic function and a suspected LVO (Supplementary Video 2). Continuous positive airway pressure was accomplished. The boy was intubated and brought to a pediatric cardiology intensive care center, where echocardiography findings were confirmed (Figure 2). Due to poor contractility and thin wall thickness in the LVO, it was determined to be an aneurysm. Intravenous treatment with milrinone and levosimendan infusion was administered. Cordarone was given due to frequent ventricular ectopic beats, with successive normalization. Treatment with digoxin and eventually also metoprolol was needed due to myocardial dysfunction. The boy was extubated at 2 days of age and stabilized with a successive improvement of the left ventricular function (Figure 2). Initial profound hyponatremia was corrected and later normalized without further treatment. No metabolic disorder was found and virology tests (PCR against parvovirus, cytomegalovirus, epstein barr virus, enterovirus, human herpesvirus) were all negative. NT-pro-BNP decreased significantly during the course from >35,000 at admission to 5,860 at the time of discharge and was normalized (175) at follow-up at 5 months of age. With the intensive medical treatment, the heart function seemed to return to normal, and eventually, all medication was terminated. At follow-up at 4 years of age, however, echocardiography still displayed low contractility and the CMR images displayed an aneurysm-like structure (ALS) inferior to the left ventricle, which was determined to be an aneurysm (Figure 2). Surgical treatment of the LVO was then discussed to prevent complications such as thrombosis, rupture of the aneurysm, and left ventricular failure. To the conventional CMR examination, a CMR 4Dflow examination was added. Based on the CMR 4Dflow examination, a thorough evaluation of the function of the left ventricle as well as the LVO was possible and the LVO was classified as a double-chambered left ventricle with almost complete blood emptying (Figure 2 and Supplementary Video 2). The clinical decision-making was assisted by this additional information and surgery was not performed. When followed up at 7 years of age, the boy was still thriving and without any medication.

## Diagnostic assessment

### Echocardiography

The patient underwent 10 echocardiographic examinations in the 3 weeks he was treated in the pediatric cardiology intensive care center at the Queen Silvia Children’s Hospital in Gothenburg. The protocol used was the standard pediatric echocardiogram according to current guidelines (6), with subcostal, parasternal, apical, and suprasternal views and including an assessment of the LVO. The examinations were performed by experienced pediatric cardiologists or clinical physiologists/technicians using General Electric Vivid E9 (GE Health Medical, Horten, Norway). At the first echocardiography examination, in addition to left ventricular dilatation, an LVO was detected without any sign of a thrombus (Figure 2). The LVO was initially evaluated as an aneurysm due to the reduced thickness and movement of the wall of the outpouching. At the following echocardiography examinations, the contractility was still low and no re-evaluation of the LVO was considered (Figure 2).

### CMR examinations

**Figure 3:**
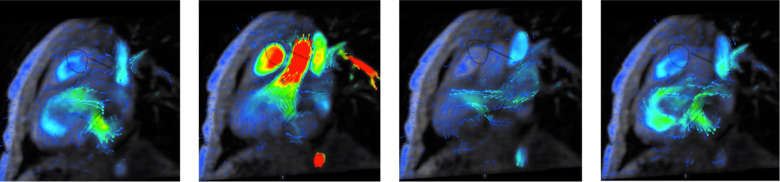
CMR 4Dflow visualization of the heart and blood flow in the 4-year-old boy. The blood flow pattern was shown to be associated with a double-chambered left ventricle with good contraction and emptying of blood from the structure but without effect on the elliptical shape or function of the main left ventricle. The clinical decision-making was facilitated by this information, and with an almost complete emptying, there was no need for operation at this juncture. Hence, 4Dflow was of significant diagnostic value in this patient. CMR, cardiac magnetic resonance.

CMR was performed at 2 weeks and 4 years of age. Both CMR examinations were performed in full anesthesia using a clinical 3T MR scanner (750 W, GE Medical Systems, Waukesha, WI, United States). The imaging protocols included bSSFP (FIESTA) and LGE sequences in breath hold, following the SCMR guidelines (7), as well as perfusion imaging in free breathing. At the second examination, at 4 years of age, 4Dflow was also added to the scan protocol to visualize the hemodynamical blood flow patterns within the heart (Figure 3 and Supplementary Video 2). An ECG-gated, free breathing, and motion-corrected 4Dflow sequence with contrast enhancement: 1 mmol/kg gadolinium (Dotarem 279.3 mg/ml, Guerbet, France) was used with the following scan parameters: TR = 4.1 ms, TE = 2.1 ms, VENC = 200 cm/s, BW = 62.5 kHz, flip angle = 8°, voxel size 2 mm × 2 mm × 2 mm. All post-processing and measurements were performed using a standalone advantage windows workstation with Cardiac VX software (GE Medical Systems), except the 4Dflow measurements, which were post-processed using clinical software from Arterys (Arterys Inc., San Francisco, CA, United States). Before implementation of the present 4Dflow sequence into the clinical practice, it was validated on 10 patients with CHD, displaying high image quality and coherence in peak flow velocity and flow volume measurements with the conventional 2Dflow method (CV_peak _< 0.2% and CV_flow _< 10%; linear regression: 4Dflowpeak = 2Dflowpeak + 2.5 with R = 0.97 and 4Dflowflow = 0.9 × 2Dflowflow − 0.3 with *R* = 1). The CMR images enabled clear views of the aneurysm-like structure, which in combination with echocardiography initially was determined to be an aneurysm with low contractility (Figure 2). The perfusion and LGE images did not detect any abnormalities within the myocardium of the aneurysm-like structure neither at the initial CMR at 14 days of age nor the 4-year follow-up. With the added CMR 4Dflow measurements, the blood flow could be visualized in detail and the structure could be classified as a double-chambered left ventricle with sufficient contraction and emptying of the blood without an effect on the elliptical shape or function of the main left ventricle (Figure 3 and Supplementary Video 2).

### Therapeutic intervention

No therapeutic intervention in addition to the initial heart failure treatment was necessary.

### Follow-up and outcomes

The boy is followed up regularly by his pediatric cardiologist using echocardiography, but no further CMR 4Dflow measurements have been performed. He is doing well without any medication.

## Discussion

There are few reports and studies in the literature describing the value of CMR 4Dflow for CHD and, to our knowledge, no previous study reports the value of the method for evaluation of LV outpouchings. In the case presented here, the initial diagnosis was aneurysm, as it presented left ventricular dysfunction on echocardiography and thin, hypokinetic wall on CMR images. With an initial clinical presentation of heart failure immediately after birth, the LVO in the present case could be either a congenital defect, a result of a prenatal event leading to myocardial stunning or even myocarditis. As the virology was negative, virus-induced myocarditis was less likely. There are no prenatal scans saved and, therefore, we cannot determine for certain whether the condition was preexisting. The preserved elliptical shape in the left ventricle at follow-up CMR, in addition to a normal wall thickness in relation to its size and a normal contraction of the LVO, suggests a congenital double-chambered left ventricle, in accordance with the classification in the study by Malakan Rad et al. (Figure 1). However, the fact that the ventricular function, as well as the contraction in the LVO, improved quickly after birth (and treatment) supports an alternative theory that the cause of the LVO was a localized cardiovascular event. This, however, is somewhat contradicted by the fact that no abnormalities were found on perfusion or LGE images at follow-up CMR, but it cannot be completely ruled out, especially considering the initial stunning (Supplementary Video 1).

Unlike echocardiography and CMR, CMR 4Dflow offers quantitative hemodynamic parameters such as wall shear stress, pulse-wave velocity, pressure difference, turbulent kinetic energy, and eccentricity (1), which may improve the decision-making and risk assessment of patients with CHD (8). Most importantly, 4Dflow can visualize complex hemodynamic blood flow patterns in terms of streamlines and vortex flow (1). Present findings show that such patterns can display new important information about heart function (Figure 2).

According to our experience, the diagnosis of congenital ventricular outpouchings can be suspected by echocardiography and confirmed by contrast-enhanced CT or CMR (5). Based on the combination of echocardiography and CMR examination, the LVO was determined to be an aneurysm and as such was planned to be corrected by a surgical operation to prevent future complications such as thrombosis, rupture of the aneurysm, and left ventricular failure. In a follow-up CMR examination, with improved visualization of the LVO by 4Dflow, the blood flow pattern was shown to be associated with a double-chambered left ventricle with good contraction and emptying of blood from the structure but without effect on the elliptical shape or function of the main left ventricle, and as such, determined to have a good prognosis (5). The clinical decision-making was facilitated by this information, and with an almost complete emptying, there was no need for operation at this juncture. Hence, 4Dflow was of significant diagnostic value in this patient.

In conclusion, we demonstrate the clinical usability of CMR 4Dflow in a 4-year-old boy with severe neonatal heart failure. The 4Dflow measurement improved the diagnostic value of the CMR examination and revealed novel hemodynamic flow patterns, facilitating the classification of the double-chambered left ventricle with no need for operation. Hence, we recommend preoperative 4Dflow visualization in children with heart failure for improved decision-making and risk assessment.

## Data Availability

The raw data supporting the conclusions of this article will be made available by the authors at request.
